# Stereoselective Asymmetric Syntheses of Molecules with a 4,5-Dihydro-1*H*-[1,2,4]-Triazoline Core Possessing an Acetylated Carbohydrate Appendage: Crystal Structure, Spectroscopy, and Pharmacology

**DOI:** 10.3390/molecules29122839

**Published:** 2024-06-14

**Authors:** Anwaar S. Al Maqbali, Nawal K. Al Rasbi, Wajdi M. Zoghaib, Nallusamy Sivakumar, Craig C. Robertson, Musa S. Shongwe, Norbert Grzegorzek, Raid J. Abdel-Jalil

**Affiliations:** 1 Department of Chemistry, College of Science, Sultan Qaboos University, Al-Khod 123, Muscat P.O. Box 36, Oman; anwaaralmaqbali94@gmail.com (A.S.A.M.); nrasbi@squ.edu.om (N.K.A.R.); zoghaibw@squ.edu.om (W.M.Z.); musa@squ.edu.om (M.S.S.); 2 Department of Biology, College of Science, Sultan Qaboos University, Al-Khod 123, Muscat P.O. Box 36, Oman; apnsiva@squ.edu.om; 3 Department of Chemistry, University of Sheffield, Sheffield S3 7HF, UK; craig.robertson@sheffield.ac.uk; 4 Institute of Organic Chemistry, University of Tübingen, Auf Der Morgenstelle 18, A-Bau, 72076 Tübingen, Germany; norbert.grzegorzek@uni-tuebingen.de

**Keywords:** asymmetric synthesis, stereoselectivity, 1,2,4-triazoline, glucopyranoside moiety, single-crystal X-ray analysis, spectroscopic techniques, anti-cancer, anti-microbial

## Abstract

A new series of chiral 4,5-dihydro-1*H*-[1,2,4]-triazoline molecules, featuring a β-ᴅ-glucopyranoside appendage, were synthesized via a 1,3-dipolar cycloaddition reaction between various hydrazonyl chlorides and carbohydrate Schiff bases. The isolated enantiopure triazolines (**8a**–**j**) were identified through high-resolution mass spectrometry (HRMS) and vibrational spectroscopy. Subsequently, their solution structures were elucidated through NMR spectroscopic techniques. Single-crystal X-ray analysis of derivative **8b** provided definitive evidence for the 3-D structure of this compound and revealed important intermolecular forces in the crystal lattice. Moreover, it confirmed the (*S*)-configuration at the newly generated stereo-center. Selected target compounds were investigated for anti-tumor activity in 60 cancer cell lines, with derivative **8c** showing the highest potency, particularly against leukemia. Additionally, substituent-dependent anti-fungal and anti-bacterial behavior was observed.

## 1. Introduction

Chirality is of paramount importance in drug design and synthesis considering that it affects the binding affinity and interactions between a drug and its target bio-receptor, thereby shaping the pharmacology of the drug. Enantiomerically or diastereomerically pure compounds can be obtained classically via chiral resolution, utilizing chiral auxiliaries, employing an enantiomerically pure substrate, a reagent, a solvent, or a catalyst as starting material [1]. The strategy relies on the use of inherited chiral topology, aiming towards the enantioselective synthesis of heterocycles, which offers a direct approach for chiral heterocycles in high yields [2,3]. Carbohydrates are ideal chiral auxiliaries because they are readily available and highly functionalized compounds with several stereogenic centers. The literature has witnessed a number of examples of enantiomerically pure natural products synthesized using carbohydrates as chiral scaffolds [4].

1,2,4-triazole derivatives such as triazolines are common pharmacophores in many drugs due to their versatile biological behavior, exhibiting activities such as anti-bacterial [5], anti-fungal [6,7], anti-tubercular [8], and anti-tumor [9] activities. In addition, the 1,2,4-triazole moiety can bring about various non-covalent interactions that influence lipophilicity and the binding ability to a biomolecular target [10]. Specifically, 1,2,4-triazole has been reported to interact strongly with heme iron while the aromatic substituents on the triazole are very effective in interacting with the active site of the aromatase enzyme [11,12]. Several compounds containing this nucleus, such as fluconazole and ribavirin, have already been developed as pharmaceutical products [13].

Widely, the synthesis of 1,2,4-triazolines has followed various pathways, each presenting its own strengths and weaknesses. Conventional methods have relied on raw materials such as amidines, imidates, amidrazones, aryldiazoniums, and hydrazones to furnish the necessary nitrogen atoms for triazole synthesis. However, reported yields have often been affected by issues such as selectivity, functional group compatibility, chirality, enantiopurity, or the complexity of precursors [14].

**Scheme 1:**
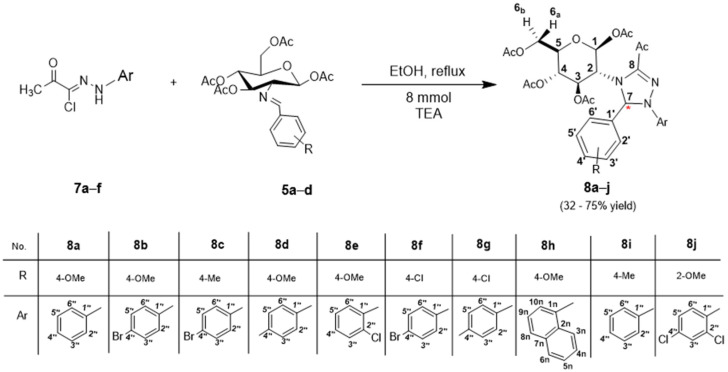
Asymmetric synthesis of 2-(3-acetyl-1-(substituted-phenyl)-5-(substituted-phenyl)-1,2,4-triazolo-4-yl)-2-deoxy-1, 3,4,6-tetraacetyl-β-ᴅ-glucose (**8a**–**j**). The new chiral stereo-center is indicated with an asterisk mark.

Herein, we report the asymmetric synthesis of a new series of 2-(3-acetyl-1-(substituted-phenyl)-5-(substituted-phenyl)-1,2,4-triazolo-4-yl)-2-deoxy-1,3,4,6-tetraacetyl-β-ᴅ-glucose derivatives (**8a**–**j**) using acetylated glucose Schiff bases as chiral scaffolds (Scheme 1). All these 4,5-dihydro-1*H*-[1,2,4]-triazoline derivatives were identified through HRMS and characterized through NMR spectroscopy. In this work, detailed structural analysis of only derivative **8b** is conducted. Comprehensive crystallographic characterization accompanied by molecular mechanics involving some of the other members of this series will be published elsewhere. Finally, selected 4,5-dihydro-1*H*-[1,2,4]-triazoline derivatives were investigated for anti-tumor and anti-bacterial activities.

## 2. Results and Discussion

### 2.1. Chemical Synthesis

**Scheme 2:**
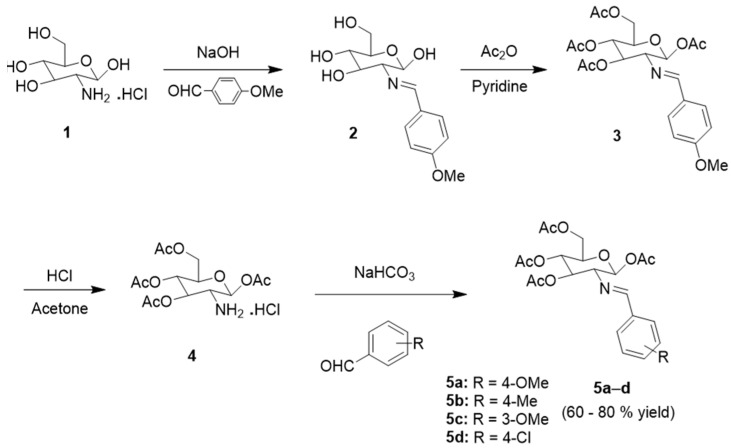
Representation of the synthetic route to the glucopyranose Schiff bases (**5a**–**d**).

The synthetic route to 2-(3-acetyl-1-(substituted phenyl)-5-(substituted phenyl)-1,2,4-triazolo-4-yl)-2-deoxy-1,3,4,6-tetraacetyl-*β*-ᴅ-glucose (**8a**–**j**) is shown in Scheme 1. The carbohydrate Schiff bases (**5a**–**d**) were coupled with the hydrazonyl chlorides (**7a**–**f**) via a 1,3 dipolar cycloaddition reaction (1,3-DCPA). The ᴅ-glucopyranose Schiff bases (**5a**–**d**) were synthesized from the commercially available ᴅ-glucosamine using an established procedure [15]. This synthesis was achieved by treating glucosamine hydrochloride with *p*-anisaldehyde in a basic medium, aiming to protect the amine group, followed by acetylation under dry and inert conditions using an excess amount of dry acetic anhydride in dry pyridine as a solvent to obtain 2-amino-2-deoxy-1,3,4,6-tetra-*O*-acetyl-*β*-ᴅ-glucopyranose (Scheme 2). The deprotection of the imine group was achieved using concentrated hydrochloric acid to free the reactive amino group. The condensation of the latter tetraacetyl glucosamine hydrochloride with variously substituted aromatic aldehydes afforded the corresponding desired Schiff bases (Scheme 2). The structures of these Schiff bases were characterized through spectroscopic techniques. 

**Scheme 3:**
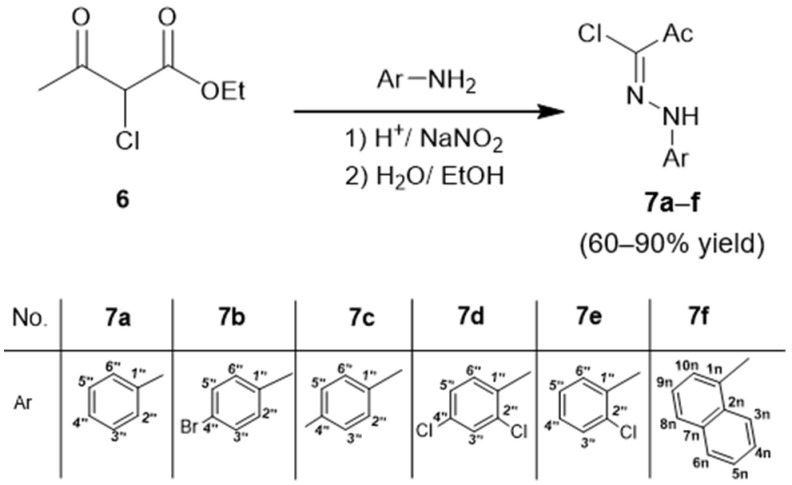
General synthetic route to the hydrazonyl chloride series (**7a**–**f**).

The hydrazonyl chlorides were prepared by coupling the respective arenediazonium salts with 3-chloro-2,4-pentanedione via the Japp–Klingemann reaction according to a reported procedure [16] as depicted in Scheme 3.

**Scheme 4:**
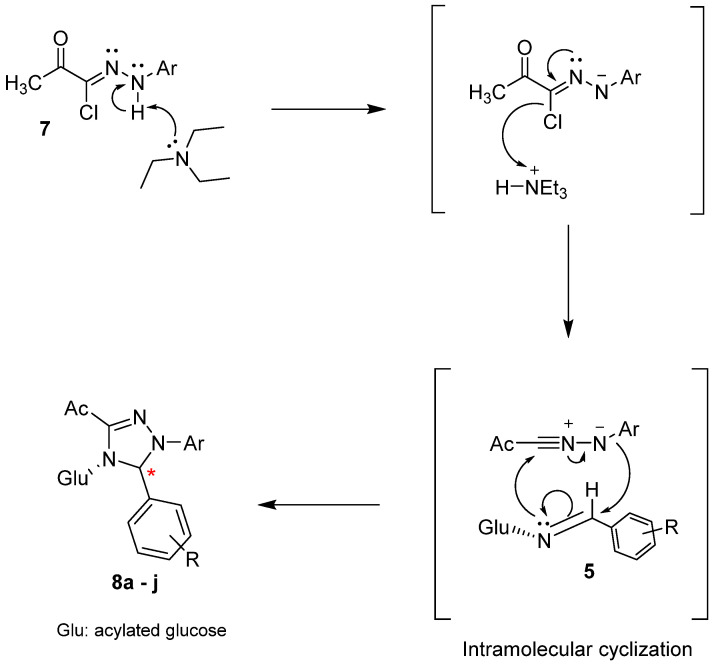
Proposed mechanism for the asymmetric synthesis of 4,5-dihydro-1*H*-[1,2,4] triazoline derivatives showing the newly generated chiral center (indicated with an asterisk mark).

The asymmetric 1,3-DPCA reaction of hydrazonyl chlorides **7a**–**f** with Schiff bases **5a**–**d** was carried out in refluxing ethanol or THF for several hours or in dichloromethane at ambient temperature. The chemical identities of the final products were ascertained through HRMS and vibrational spectroscopy. The solution structures were characterized through NMR spectroscopy. The solid-state structure of **8b** was determined through single-crystal X-ray analysis, which revealed the configuration at the new stereo-center. The 1,3-DPCA reaction is a powerful tool for the construction of enantiomerically or diastereomerically pure five-membered heterocycles. The proposed mechanism for the asymmetric synthesis of the target compounds is illustrated in Scheme 4. This reaction begins with the formation of a nucleophilic nitrile imine from the hydrazonyl chloride via deprotonation using triethylamine (TEA) as a base, followed by the nucleophilic addition of the nitrile imine intermediate to the electrophilic glucopyranose Schiff bases. Subsequently, the intramolecular cyclization leads to the corresponding five-membered ring. It is noteworthy that although a similar synthetic route to 1,2,4-triazoline derivatives via 1,3-DPCA is known and has been described in the literature [17,18], the synthetic pathway presented in this work is the first example to construct a triazoline ring on a sugar moiety.

The absolute configuration (*R*/*S*) of the newly generated stereo-center at C-7 (Scheme 1) in the structures of the target triazoline derivatives was unequivocally determined in the case of six compounds in this series. The absolute configuration of C-7 was assigned as (*S*) in compounds **8b**, **8c**, and **8f** but as (*R*) in compounds **8a**, **8g**, and **8i**. Unfortunately, the X-ray structures of the remaining derivatives **8d**, **8e**, **8h**, and **8j** could not be determined as their crystals were not suitable for X-ray diffraction. The crystallographic analyses of derivatives **8a**–**c**, **f**, **g**, and **i** revealed that only one diastereomer had been isolated via crystallization. This may be explained in terms of the differences in intermolecular forces, such as H-bonding, which could cause disparities in the solubilities between the two diastereomers. In this work, the structural analysis focuses on derivative **8b**. A detailed crystallographic characterization of the other derivatives, along with DFT calculations, will be communicated elsewhere.

### 2.2. ^1^H NMR and ^13^C NMR Spectroscopic Characterization

The ^1^H NMR spectra of the 4,5-dihydro-*1H*-[1,2,4]-triazoline derivatives showed the aromatic protons in the range of 7.40–6.60 ppm while the five acetyl–CH_3_ protons appeared upfield as five singlets. In the scheme, the presence of a singlet peak at around 6.00 ppm indicates the occurrence of the new stereo-center (**C**-**7**). The anomeric proton (**1**) resonates at around 6.50–6.62 ppm as a doublet with a coupling constant of ~8 Hz. The signals for protons **2**–**5** appear as doublet-of-doublets (dd) or a quasi-triplet (qt) due to the overlapping of the peaks in some cases with coupling constants at around 8–10 Hz. Extensive NMR analysis of the triazoline derivatives demonstrated clearly that all members of this series had the ^4^C_1_ chair conformation since J_1,2_ and J_2,3_ values were high (7–9 Hz), indicating a di-axial relationship. ^13^C NMR spectroscopy confirmed the structures of the compounds in solution. The ^13^C NMR spectra displayed similar signals for all the compounds in this series, which were assigned to the ᴅ-glucopyranose carbons, carbonyl carbons, and acetyl carbons. However, the aromatic carbons showed slight differences in the chemical shifts depending on the substituents. Detailed information is provided in the Experimental Section, Section 3.7.

**Figure 1:**
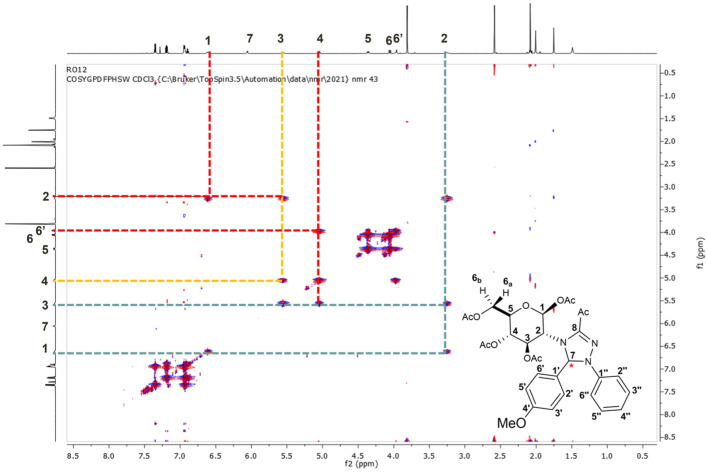
COSY NMR spectrum for compound **8a** in CDCl_3._ the newly generated chiral center (indicated with an asterisk mark).

Extensive NMR analysis of the products was investigated further through 2D NMR spectroscopic methods such as COSY, HMBC, and HSQC. In Figure 1, the COSY NMR spectrum for one of the derivatives (**8a**) shows the correlation between the protons, which is consistent with the labelled protons in the structure (Scheme 1). However, the absence of any correlation with proton **7** provides strong evidence for the existence of the expected chiral center. The HSQC and HMBC NMR spectra of the same derivative (**8a**) are provided in the Supplementary Materials (Figures S1 and S2, respectively).

### 2.3. Crystallographic Analysis of Derivative ***8b***

**Figure 2:**
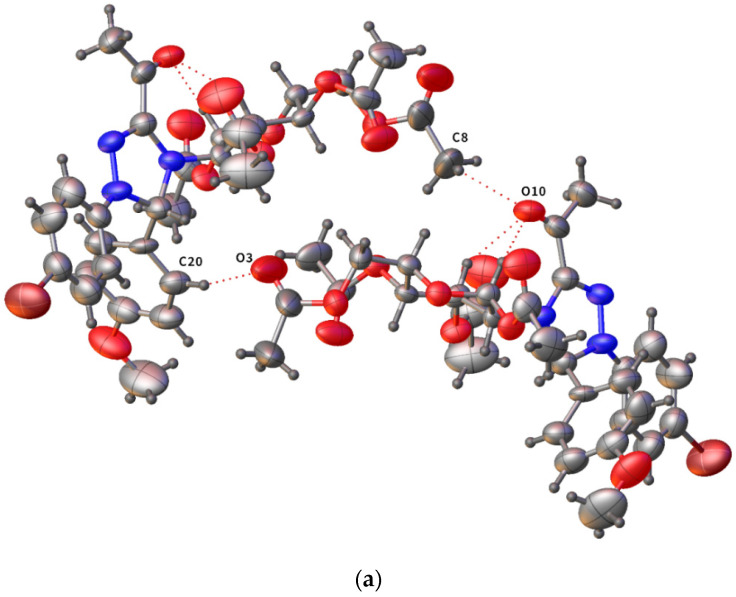

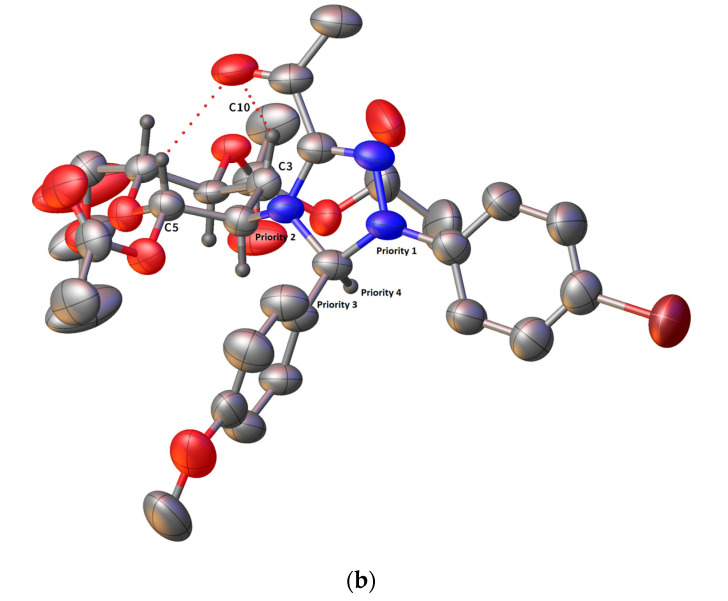
(**a**) Non-covalent interactions of **8b** in the unit cell; (**b**) asymmetric unit and assignment of absolute configuration.

The 3-D structure of derivative **8b** was determined definitively through single-crystal X-ray crystallography. A lustrous yellow block of this compound was employed for X-ray diffraction; intensity data were collected at room temperature. The crystal data and parameters for data collection and structure refinement are presented in Table 1. Derivative **8b** crystallized in the monoclinic chiral *P*2_1_ space group with *Z* = 2 and one molecule in the crystallographic asymmetric unit (Figure 2). The two identical molecules in the unit cell were linked through two weak non-classical intermolecular hydrogen-bonding interactions [(–CH_2_CO_2_Me) C(8)–H···O(10) (–COMe)] and [*p*-OMe-phenyl) C(20)···O(3) (–CO_2_Me)] to form a stable dimer (Figure 2a, Table 2). The glucopyranoside moiety, adopting a chair conformation, is intramolecularly hydrogen-bonded to the carbonyl oxygen atom of the acetyl group on the triazoline ring [C(3)–H···O(10), C(5–H···O(10)] Figure 2b, Table 2. The packing diagram shows **8b** molecules weakly interacting through H-bonding [–CO_2_Me) C(12)···Br(1)] to form a 1D chain along the *b* axis, which is the screw axis of the crystal structure (Figure S3, Table 2). 

**Figure 3:**
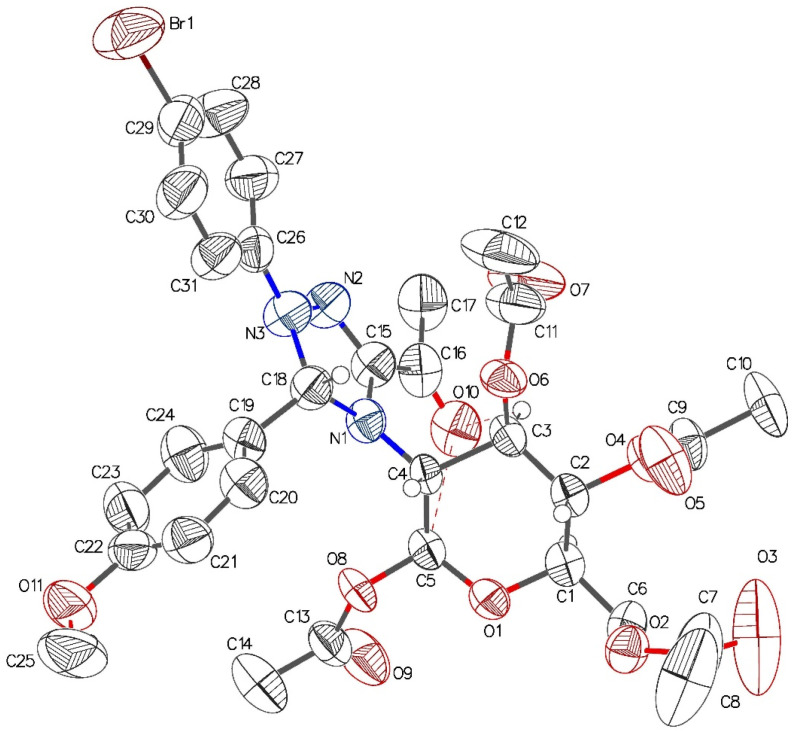
ORTEP diagram for the molecular structure of derivative **8b** with the atom-numbering scheme.

The molecular structure of **8b** is depicted in Figure 3. As can be seen from the perspective view, the structure of this molecule consists of a planar triazoline ring bearing acetyl, *p*-bromophenyl, and *p*-methoxyphenyl substituent groups along with a tetra-*O*-acetyl-β-ᴅ-glucopyranoside appendage. Derivative **8b** is chiral, as are the rest of the molecules in this series, and C(18) of the triazoline ring is the stereogenic center with an *S*-configuration (Figure 2b).

The length of C(15)–N(2) [1.299(12) Å] (Table 3) is consistent with a double-bond character and lies within the range of literature values (1.27–1.32 Å) for triazolines and triazoles [19,20,21,22,23,24,25,26]. As expected, the adjacent C–N bond [C(15)–N(1) = 1.415(13) Å] is longer by virtue of being a σ-bond. However, it is somewhat shorter than C(18)–N(1) [1.490(12) Å] due to the differences in the orbital hybridizations of C(15) and C(18) (sp^2^ and sp^3^, respectively). The distance of N–N [N(2)–N(3) = 1.361(11) Å] is normal and compares favorably with those observed in other triazoline and triazole cores (1.36–1.42 Å) [19,20,21,22,23,24,25,26].

The acetyl (Ac) and four acetyloxy (OAc) groups of **8b** have carbonyl bond distances (Table 3) within the range reported in the literature (1.17–1.19 Å) [24,26], with the exception of C(13)–O(9) [1.217(14) Å], which is weakened by its involvement in intermolecular H-bonding [C(10)–H(10B)···O(9)] (Table 2). Consistent with C(sp^2^)–O(sp^3^) σ-bonding, the OAc C–O single bonds are longer with characteristic distances in the range 1.340–1.358 Å [25,27]. Given that bromine is the largest atom in this molecule, its covalent bond C(29)–Br(1) is the longest [1.887(13) Å] and is comparable with those encountered in closely related compounds [24,26].

### 2.4. FTIR Spectroscopy

**Figure 4:**
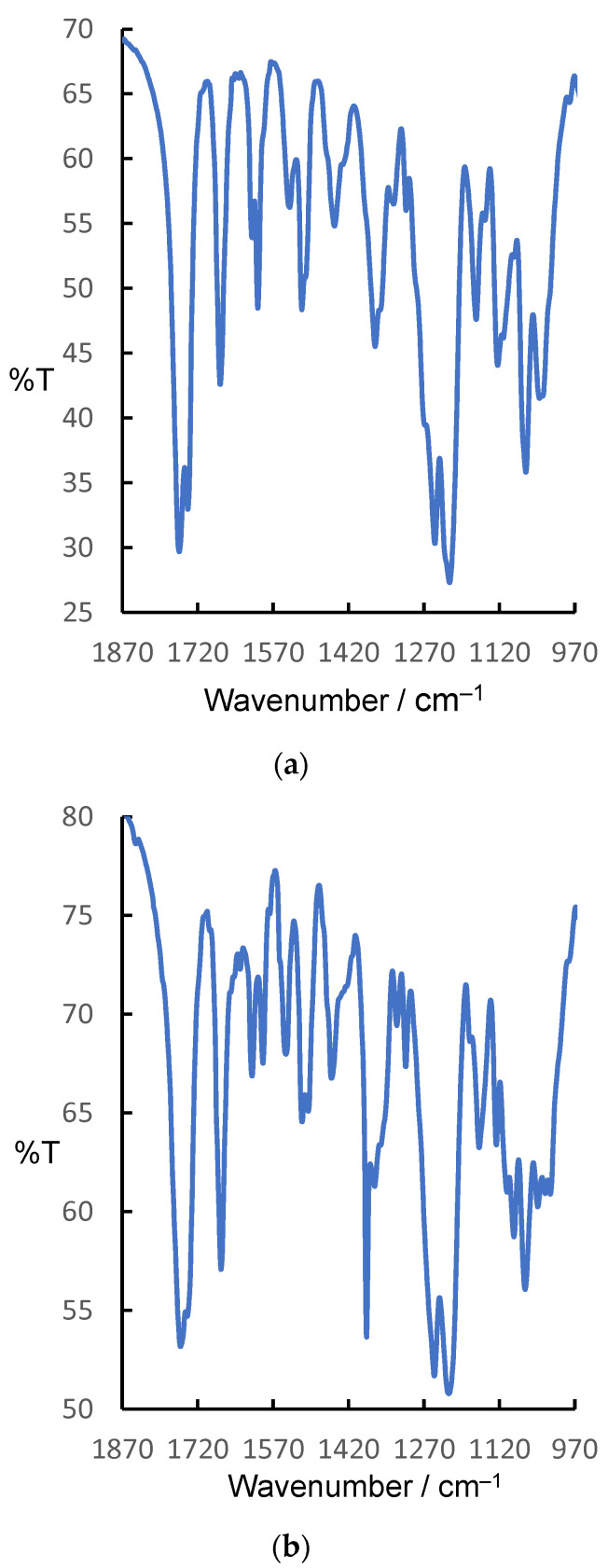
A portion of the IR spectra (1870–970 cm^–1^) of derivatives (**a**) **8a** and (**b**) **8b**.

The functional groups and other structural features of interest in derivatives **8a**–**8j** were identified through IR spectroscopy. The IR spectra of derivatives **8a** and **8b**, representative of those of the other members in this series, are presented in Figure 4; the complete spectrum (4000–400 cm^–1^) of **8a** is displayed in Figure S4. For all 10 derivatives, selected pertinent IR data are provided in Section 3.7. Figure 4 highlights the features that these compounds have in common.

As can be seen from Figure 4, the vibrational absorption bands of the ester (–OCOMe) and acetyl (–COMe) groups are conspicuous and recognizable not only by their huge intensities but also by their characteristic stretching frequencies. The carbonyl (C=O) bond and the associated C–O bond of the acetyloxy group occur as intense absorptions in the ranges 1756–1727 and 1223–1211 cm^–1^, respectively. The other signature feature of derivatives **8a**–**8j** is the acetyl group attached to the triazoline ring whose carbonyl vibration is evidenced by the intense absorption band in the range ~1655–1676 cm^–1^. These wavenumbers are somewhat lower than expected, presumably on account of the weakening of this multiple bond due to the involvement the carbonyl oxygen atom in H-bonding interactions as revealed by the X-ray structural analysis of **8b** (Figure 2 and Figure 3 and Table 2).

The IR absorption with a stretching frequency between 1600 and 1613 cm^–1^ is attributable to the ν(C=N) of the triazoline ring. The aromatic carbon–carbon stretches occure in the range 1440–1600 cm^–1^; the aromatic and aliphatic C–H stretches were observed in the usual regions 3000–3100 cm^–1^ and 2840–2990 cm^–1^, respectively. Finally, the ν(C–O), ν(C–N), and ν(N–N) values are comparable with those reported in the literature [15,28,29,30,31,32,33,34] for closely related molecules. 

### 2.5. Biological Assay

#### 2.5.1. Anti-Tumor Activity

Compounds **8a**–**f** were selected by the National Cancer Institute (NCI/NIH, USA) for a One-Dose Screen test at a single high dose against mainly nine panels, namely leukemia, non-small cell lung cancer, colon cancer, CNS cancer, melanoma cancer, ovarian cancer, renal cancer, prostate cancer, and breast cancer. The results were reported as a mean graph of the percent growth (G%) of treated cells. The total mean growth percentage of cancer tumor cell lines for all submitted samples is presented in Table 4.

The mean growth of treated tumor cell lines against all submitted compounds (Table 4) revealed that **8c** and **8f** show the lowest mean G% values, meaning they had the highest anti-cancer activity compared to the other selected compounds. Compound **8c** showed maximum activity against some cancer cell lines. From the mean growth plot of **8c**, the highest growth inhibition was shown in the leukemia cell line (HL-60(TB): 14.42%). In addition, it represented a significant reduction in the CNC cancer cell line (SF-295: 29.43%) and breast cancer cell line (T-47D: 26.78%). However, this derivative exhibit weak results in colon cancer, melanoma cancer, ovarian cancer and non-small cell lung cancer, observed by growth percentage mostly above 50%.

It is noteworthy that compounds **8c** and **8f** exhibited activity in the same panel/cell lines (shown in Table 5).

#### 2.5.2. Anti-Bacterial Activity

**Figure 5:**
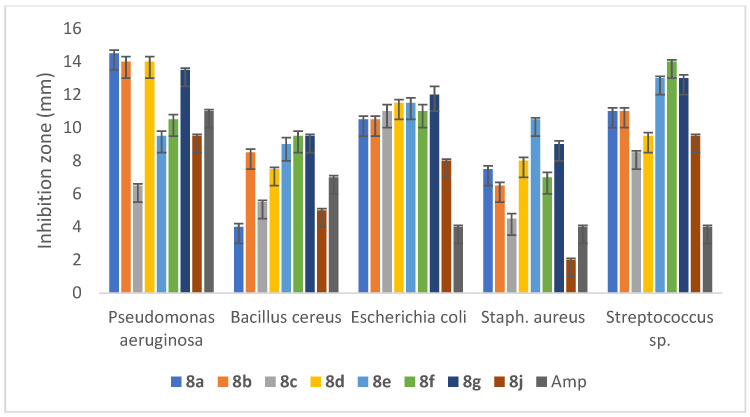
Anti-bacterial activity results as inhibition zones for tested compounds (80 μL of 2.0–2.5 mg/mL of different samples was used per disc). The results are expressed as mean values ± SDs (n = 3).

Anti-bacterial results were interpreted in terms of the diameter of the inhibition zone along with the positive control Ampicillin 10 μg (AMP 10) against five different bacterial cultures, namely, *Staphylococcus aureus*, *Escherichia coli*, *Pseudomonas aeruginosa*, *Bacillus cereus*, and *Streptococcus* spp. The anti-bacterial activity of different compounds is presented in Figure 5. Practically, when the Mueller–Hinton medium was used without any supplements, no inhibition zone was observed due to the diffusion difficulties of the compounds that tended to crystallize on the surface of the medium while, when nutrient agar or plate count agar was used, the results were observed clearly. The highest anti-bacterial activity of the derivatives was found against *P. aeruginosa*, followed by *Streptococcus* sp. and *E. coli*, with different satisfactory potential values. The largest zones of growth inhibition against *P. aeruginosa* were observed for the compounds **8a** (12.5 mm), **8b** (12 mm), **8d** (11.5 mm), and **8g** (13.5 mm). Interestingly, the activity of these four derivatives was higher than the inhibition activity of the positive control (AMP 10), with a difference of 1.5–3.5 mm. In general, the inhibition activity of the investigated series decreased against *S. aureus* and *B. cereus.* From the results, we observe that the compounds examined exhibit higher anti-bacterial activity when halogens such as chlorine or bromine are present. According to a structure–activity relationship analysis of 1,2,4-triazoline Schiff bases, the 4-hydroxy-3-methoxyphenyl moiety at the *N*-4 position and the nitro group in the phenyl ring at the 3-position were both essential for the 1,2,4-triazoline to exhibit a strong anti-bacterial effect [35]. The docking studies published previously for similar 1,2,4-triazoline derivatives provided some possible inhibition mechanisms. These docking results reveal a possible inhibitory potential against glucosamine-6-phosphate synthase (an enzyme involved in N-acetylglucosamine synthesis, which is an essential building block of both bacterial and fungal cell walls), DNA gyrase (a type II topoisomerase that can introduce negative supercoils into DNA), SecA ATPase (which drives the post-translational translocation of proteins through the SecY channel in the bacterial inner membrane), and dihydrofolate reductase (which catalyzes the NADPH-dependent reduction of dihydrofolate to tetrahydrofolate, the precursor of DNA synthesis), which are indispensable enzymes for bacterial function [36].

#### 2.5.3. Anti-Fungal Activity

**Figure 6:**
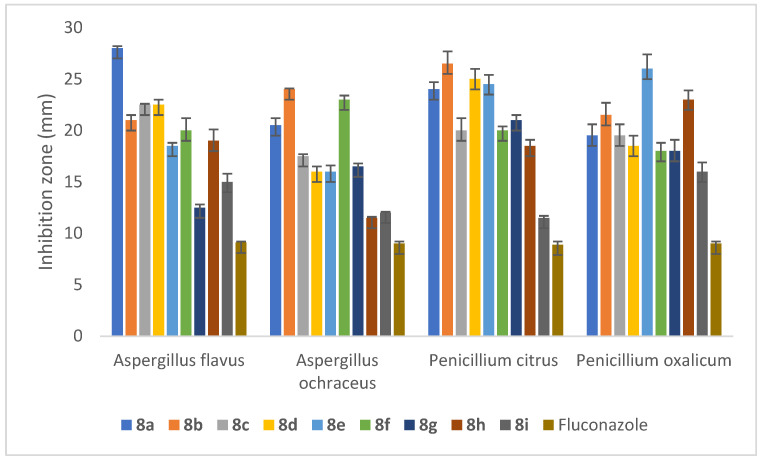
Anti-fungal activity results as inhibition zones for tested compounds (100 µg of each compound was used per well). The results are expressed as means ± SDs (*n* = 3).

To study the anti-fungal activity of our compounds, the following fungi were used: *Aspergillus flavus*, *Aspergillus ochraceus*, *Penicillium citrus*, and *Penicillium oxalicum*. The results of the anti-fungal activities illustrated in Figure 6 showed that all the compounds were active against all the fungi tested.

Among the compounds tested, the halogen-substituted triazolines demonstrated promising anti-fungal activity, with **8b** and **8e** showing the highest activity against *Penicillium* fungi. However, compounds **8a** and **8d** (with a methoxy substituent group) showed better activity than others such as **8i** and **8g**. In general, the anti-fungal efficiency of 1,2,4-triazolines appears to be due to their methoxy and halogen substituents.

**Figure 7:**
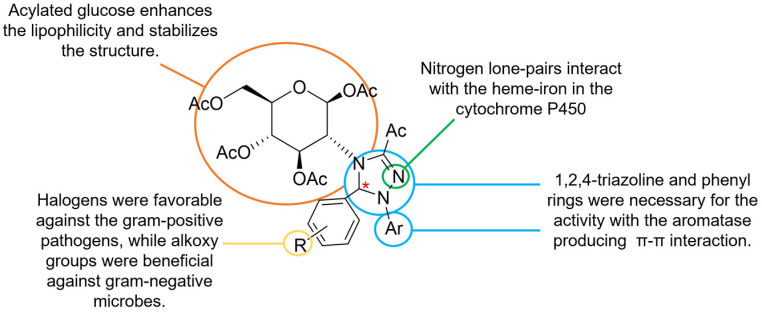
General chemical structure of the 1,2,4-triazoline derivatives and the predicted biological activity sites [42,43,44]. The asterisk mark represents the newly generated chiral center.

Based on the literature survey of the biological activity of anti-fungal triazolines, they may act by inhibiting the cytochrome P450, which is a key enzyme in the biosynthesis of ergosterol from lanosterol, which is helpful in cell wall synthesis [37]. Triazolines bind to the active site of cytochrome P450 14-demethylase via a combination of heme coordination, hydrophobic interactions, and hydrogen bonding [38,39,40]. The proposed mechanism behind this inhibition is that the basic nitrogen of the triazoline ring is binding tightly to the heme-iron of the fungal cytochrome P450, blocking substrate and oxygen binding. The inhibition of the 14α-demethylase results in the accruing of sterols, leading to permeability changes and the malfunctioning of membrane proteins [41]. Overall, the predicted biological active sites are summarized in Figure 7. 

## 3. Experimental Setup

### 3.1. General

Reagents and solvents were purchased from Sigma-Aldrich and used without further purification. Thin layer chromatography (TLC) analysis was performed on glass plates pre-coated with silica gel (Whatman F 254 nm; VWR, Belfast, UK). Plates were visualized using UV GL-58 Handheld 254/365 nm UV lamp (UVP, Cambridge, UK). Flash column chromatography was performed using Kieselgel 60 (mesh 70–230) purchased from Sigma-Aldrich Ltd. (Poole, Dorest, UK). Elution for FCC usually employed a stepwise solvent polarity gradient, correlated with TLC mobility.

### 3.2. Physical Techniques of Characterization

Melting points were measured with an SMP2 Stuart Scientific apparatus. FT-IR spectra were recorded in the range 4000–400 cm^–1^ on a BRUKER Alpha ATR spectrophotometer for all derivatives and a Perkin-Elmer FT-IR spectrophotometer as KBr disks in the case of derivatives **8a** and **8b**. High-Resolution Mass Spectrometry (HRMS) was performed using a maXis mass spectrometer (Bruker Compass Data Analysis with ESI source). NMR spectra were recorded on a BRUKER ASCEND 700 MHz NMR spectrometer with a CRYO platform accessory in parts per million (ppm). CDCl_3_ was used as the solvent and tetramethylsilane (TMS) as an internal standard for calibration of chemical shifts (δ = 0). ^13^C NMR spectra were recorded on the same spectrometer at 176 MHz. All significant resonances (carbon skeleton) were assigned as per COSY (^1^H–^1^H) and HSQC (^1^H–^13^C) correlations.

### 3.3. Single-Crystal X-ray Analysis

A single yellow block of **8b** with the dimensions 0.10 × 0.10 × 0.05 mm was selected for X-ray diffraction. Intensity data were collected at room temperature using a STOE IPDS II diffractometer equipped with Mo-K_α_ radiation (λ = 0.71073 Å). The sample temperature was controlled using an Oxford Diffraction Cryojet apparatus. Data were integrated using the Stoe X-AREA software package v.1.3 [45]. The numerical absorption coefficients, μ, for MoKα radiation is 7.383. The numerical absorption correction was applied using the X-RED software package v.1.28 [46]. The data were corrected for Lorentz and Polarizing effects. The structure was solved by direct methods and subsequent differences Fourier maps and then refined on *F*^2^ via a full-matrix least-squares procedure using anisotropic displacement parameters with SHELXS-97 [47]. All the hydrogen atoms were positioned geometrically in idealized positions and refined with the riding model approximation, with *U*_iso_(H) = 1.2 or 1.5 *U*_eq_(C). All the refinements were performed using the WinGX [48] suite of programs. For the molecular graphics, the Mercury program package was used [48]. The non-hydrogen atoms were refined anisotropically. The structure has an embedded disordered hexane molecule of crystallization. The attempted solvent disorder model was not completely satisfactory, thus necessitating implementation of the PLATON SQUEEZE program [49]. The SQUEEZE calculation resulted in an electron count of 58 electrons consistent with a hexane solvent molecule. The details of the SQUEEZE calculation are included in the CIF archive file. For molecular graphics and preparation of publication materials, the program OLEX2 was used [50]. The crystallographic details of **8b** are summarized in Table 1. CCDC 2327704 contains the supplementary crystallographic data for this paper, which can be obtained free of charge from The Cambridge Crystallographic Data Centre via www.ccdc.cam.ac.uk/data_request/cif (accessed on 2 June 2024).

### 3.4. Anti-Tumor Activity

Anti-cancer screening was performed via the Developmental Therapeutics Program of the National Cancer Institute (NCI/NIH), Bethesda, MD, USA.

Developmental Therapeutics Program (DTP) of the National Cancer Institute (NCI) was initiated in April 1990. The current in vitro anti-cancer screening employed 60 human tumor cell lines representing 9 tissue types to be screened. DTP’s anti-cancer screening program is used to evaluate the efficacy of synthetic compounds and natural products [51]. All compounds submitted to the NCI 60 cell screening were tested at a single high dose against mainly nine panels. Each panel consisted of more than three cell lines where each cell line was inoculated and pre-incubated on a microtiter plate. Test agents were then added at a single concentration (1.00 × 10^−5^ M) and the cultures were incubated for 48 h [52]. One dose-response parameter (GI50) was calculated for each of the NCI 60 cell lines.

Growth inhibition was calculated relative to cells without drug treatment and the time zero control. This allowed detection of both growth inhibition (values between 0 and 100) and lethality (values less than 0).

### 3.5. Anti-Bacterial Activity

The newly synthesized compounds were screened for their anti-bacterial activities using the disc diffusion method against five different bacterial strains. These strains were obtained from Sultan Qaboos University Hospital, Muscat, Oman. These five bacteria were aseptically inoculated into nutrient broth (NB) and grown at 37 °C in the incubator. After 24 h, 1 mL of the incubated bacterial suspension was mixed with plate count agar (OXOID, Basingstoke, UK) medium and transferred to Petri plates using the pour-plate method. Filter paper discs of 5 mm (Whatman, no.3) were loaded with 80 µL of 2.0–2.5 mg/mL of different samples. The discs were allowed to remain at room temperature until complete solvent evaporation, and then, they were placed onto the agar surface. Then, the plates were incubated at 37 °C for 24 h [52]. The anti-microbial activity was determined based on the observed zones of growth inhibition (mm) of selected bacterial strains in the presence of the tested compounds. Commercial reference discs were used (*Ampicillin* 10 μg, purchased from OXOID^®^) for comparison. All tests were performed in triplicates. The stock solutions of the tested compounds were freshly prepared in dimethyl sulfoxide (DMSO).

### 3.6. Anti-Fungal Activity

Anti-fungal activity was measured via the well diffusion method against four different fungi. These fungi were inoculated into potato dextrose agar medium (PDA) and then incubated at 28 °C for 4 days. After 4 days, the fungal spores were collected and subsequently dispersed in a 20% tween-20 solution in Czapek Dox Agar (HIMEDIA, Thane, India) plates. Using an agar well borer, the wells were made, and 100 μL of 2.0–2.5 μg/mL 4,5-dihydro-*1H*-[1,2,4]-triazoline compounds was added to the wells. Then, the plates were incubated at 28 °C for 4 days. Inhibition zones were measured according to the Kanmani and Lim method (2013). The average values of the triplicate experiments were taken for the study. Anti-fungal results were interpreted in terms of the diameter of the inhibition zone (compared with the positive anti-fungal control (Fluconazol 75 μg)).

### 3.7. General Synthesis of 4,5-Dihydro-1H-[1,2,4] Triazoline Derivatives

Each of Schiff bases **5a**–**d** (1 mmol) was added to a solution of hydrazonyl chlorides **7a**–**f** (2 mmol) in 5 mL of ethanol or THF. Then, triethylamine (8 mmol) in 5 mL ethanol was added dropwise to the stirred reaction mixture. The resultant solution was heated under reflux until TLC showed the complete consumption of the Schiff base. The product was observed as an intense yellow spot on the TLC plate that fluoresced under long-wavelength UV light. Subsequently, the reaction mixture was kept at room temperature overnight to form yellow needle-shaped crystals, which were then filtered off and washed with ice-cold ethanol. In some reactions, more purification was performed via column chromatography or preparation TLC and recrystallization from hot ethanol.

#### 3.7.1. 2-(3-Acetyl-1-(phenyl)-5-((R)-4-methoxyphenyl)-1,2,4-triazolo-4-yl)-2-deoxy-1,3,4,6-tetraacetyl-β-ᴅ-glucose (**8a**)

Recrystallization of the product from hot ethanol afforded shiny yellow microcrystals (35.4% yield) with mp 146–147 °C and optical rotation: ${\left[\propto \right]}_{589}^{25}$ + 40.31(ethyl acetate).

^1^H NMR (700 MHz, CDCl_3_) δ (ppm): 7.35 (m, 2H, **2**′, **6**′), 7.19 (m, 2H, **3**″, **5**″), 6.94 (m, 4H, **2**″, **6**″, **3**′, **5**′), 6.90 (m, 1H, **4**″), 6.61 (d, *J* = 8.38 Hz, 1H, **1**), 6.06 (s, 1H, **7**), 5.56 (m, 1H, **3**), 5.05 (m, 1H, **4**), 4.36 (dd (*J* = 12.4, 4.5 Hz), 1H, **6_a_**), 4.05 (dd, *J* = 12.5 Hz, 2.1 Hz, 1H, **6_b_**), 3.97 (m, 1H, **5**), 3.81 (s, 3H, **OCH_3_**), 3.25 (m, 1H, **2**), 2.59–1.49 (5 singlets, **acetyl-CH_3_**). ^13^C NMR (176 MHz, CDCl_3_) δ (ppm): 190.8–168.6 (5 **carbonyl carbons**), 160.6 C (**4**′), 144.9 C (**8**), 142.3 C (**1**″), 131.8 C (**1**′), 129.1 CH (**2**′, **6**′), 128.6 CH (**3**″, **5**″), 121.5 CH (**4**″), 114.6 CH (**3**′, **5**′), 113.9 CH (**2**″, **6**″), 92.2 CH (**1**), 89.2 CH (**7**), 73.1 CH (**3**), 72.0 CH (**5**), 68.9 CH (**4**), 62.9 CH_2_ (**6**), 61.7 (**2**), 55.4 CH_3_ (**OCH_3_**), 26.6–20.4 (5 **acetyl-CH_3_**). IR: υ (cm^–1^): 1740, 1757, 1676 (C=O), 1215 (C–O). Theoretical HRMS (M + Na)^+^ = 648.21693, Exp *m*/*z* = 648.21682.

#### 3.7.2. 2-(3-Acetyl-1-(4-bromophenyl)-5-((S)-4-methoxyphenyl)-1,2,4-triazolo-4-yl)-2-deoxy-1,3,4,6-tetraacetyl-β-ᴅ-glucose (**8b**)

The product comprised shiny yellow needles (65% yield) with mp 140–141 °C and optical rotation: ${\left[\propto \right]}_{589}^{25}$ + 71.40 (ethyl acetate).

^1^H NMR (700 MHz, CDCl_3_) δ (ppm): 7.29 (m, 2H, **2**′, **6**′), 7.25 (m, 2H, **3**″, **5**″), 6.91 (d, *J* = 8.00 Hz, 2H, **2**″, **6**″), 6.77 (d, *J* = 8.00 Hz, 2H, **3**′, **5**′), 6.56 (d, *J* = 8.37 Hz, 1H, **1**), 5.97 (s, 1H, **7**), 5.52 (dd, *J* = 8.02, 7.99 Hz, 1H, **3**), 5.02 (dd, *J* = 8.96, 10.14 Hz, 1H, **4**), 4.33 (dd, *J* = 12.48, 4.45 Hz, 1H, **6_a_***)*, 4.02 (dd, *J* = 12.05, 2.01 Hz, 1H, **6_b_**), 3.93 (m, 1H, **5**), 3.78 (s, 3H, **OCH_3_**), 3.21 (dd, *J* = 8.00, 7.89 Hz, 1H, **2**), 2.59–1.49 (5 singlets, **acetyl-CH_3_**). ^13^C NMR (176 MHz, CDCl_3_) δ (ppm): 190.8–168.6 (5 **carbonyl carbons**), 160.6 C (**4**′), 144.9 C (**8**), 142.3 C (**1**″), 131.8 C (**1**′), 129.1 CH (**2**′, **6**′), 128.6 CH (**3**″, **5**″), 121.5 CH (**4**″), 114.6 CH (**3**′, **5**′), 113.9 CH (**2**″, **6**″), 92.2 CH (**1**), 89.2 CH (**7**), 73.1 CH (**3**), 72.0 CH (**5**), 68.9 CH (**4**), 62.9 CH_3_ (**6**), 61.7 (**2**), 55.4 CH_3_ (**OCH_3_**), 26.6–20.4 (5 **acetyl-CH_3_**). IR: υ (cm^–1^): 1757, 1740, 1674 (C=O), 1215 (C–O). Theoretical HRMS (M + Na)^+^ = 726.12744, Exp. *m*/*z* = 726.12709.

#### 3.7.3. 2-(3-Acetyl-1-(4-bromophenyl)-5-((S)-4-methylphenyl)-1,2,4-triazolo-4-yl)-2-deoxy-1,3,4,6-tetraacetyl-β-ᴅ-glucose (**8c**)

The product was purified through column chromatography to give shiny yellow microcrystals (70% yield) with mp 145–146 °C and optical rotation: ${\left[\propto \right]}_{589}^{25}$ + 62.21 (ethyl acetate).

^1^H NMR (700 MHz, CDCl_3_) δ (ppm): 7.28 (m, 4H, **2**′, **6**′, **3**″, **5**″), 7.23 (d, *J* = 7.90 Hz, 2H, **2**″, **6**″), 6.80 (m, 2H, **3′**, **5′**), 6.59 (d, *J* = 8.19 Hz, 2H, **1**), 6.01 (s, 1H, **7**), 5.56 (dd, *J* = 10.00, 9.89 Hz, 1H, **3**), 5.05 (dd, *J* = 9.50, 10.00 Hz, 1H, **4**), 4.36 (dd, *J* = 12.50, 4.50 Hz, 1H, **6_a_**), 4.05 (dd, *J* = 12.40, 2.01 Hz, 1H, **6_b_**), 3.96 (m, 1H, **5**), 3.26 (dd, *J* = 9.60, 10.00 Hz, 1H, **2**), 2.58 (s, 3H, **ph-CH_3_**), 2.36–1.42 (5 singlets, **acetyl-CH_3_**). ^13^C NMR (176 MHz, CDCl_3_) δ (ppm): 190.8–168.6 (5 **carbonyl carbons**), 145.2 C (**8**), 141.2 C (**4′**), 136.2 C (**1**″), 132.0 CH (**3**″, **5**″), 130.01 CH (**3′**, **5′**), 127.2 CH (**2**″, **6**″), 115.3 CH (**2′**, **6′**), 113.8 CH (**4**″), 92.12 CH (**7**), 73.1 CH (**1**), 72.0 CH (**5**), 68.9 CH (**4**), 65.9 CH (**3**), 62.8 CH_2_ (**6**), 61.6 CH (**2**), 27.1 CH_3_ (**ph-CH_3_**), 26.6–20.1 (5 **acetyl-CH_3_**). IR: υ (cm^–1^): 1741, 1727, 1675 (C=O), 1207 (C–O). Theoretical HRMS (M + Na)^+^ = 710.13253, Exp. *m*/*z* = 710.13169.

#### 3.7.4. 2-(3-Acetyl-1-(4-methylphenyl)-5-(4-methoxyphenyl)-1,2,4-triazolo-4-yl)-2-deoxy-1,3,4,6-tetraacetyl-β-ᴅ-glucose (**8d**)

The product comprised yellow needle-like microcrystals (65% yield) with mp 142.7–142.9 °C and optical rotation: ${\left[\propto \right]}_{589}^{25}$ + 71.70 (ethyl acetate).

^1^H NMR (700 MHz, CDCl_3_) δ (ppm): 7.34 (d, *J* = 8.73 Hz, 2H, **2**′, **6**′), 6.99 (d, *J* = 7.00 Hz, 2H, **3**″, **5**″), 6.93 (d, *J* = 8.63 Hz, 2H, **2**″, **6**″), 6.84 (d, *J* = 8.56 Hz, 2H, **3**′, **5**′), 6.62 (d, *J* = 8.36 Hz, 1H, **1**), 6.04 (s, 1H, **7**), 5.56 (dd, *J* = 7.00, 7.10 Hz, 1H, **3**), 5.05 (dd, *J* = 9.55, 10.00 Hz, 1H, **4**), 4.36 (dd, *J* = 12.40, 4.60 Hz, 1H, **6_a_**), 4.06 (dd, *J* = 12.40, 2.14 Hz, 1H, **6_b_**), 3.96 (m, 1H, **5**), 3.26 (dd, *J* = 9.65, 9.99 Hz, 1H, **2**), 3.81 (s, 3H, **OCH_3_**), 2.58 (s, 3H, **Ph–CH_3_**), 2.24–2.01 (5 singlets, **acetyl–CH_3_**). ^13^C NMR (176 MHz, CDCl_3_) δ (ppm): 170.7–168.7 (5 **carbonyl carbons**), 160.6 C (**4**′), 140.0 C (**8**), 132.3 C (**1**″), 132.0 CH (**1**′), 129.7 CH (**2**′, **6**′), 128.6 CH (**3**″, **5**″), 124.6 C (**4**″), 114.5 CH (**3′**, **5′**), 113.7 CH (**2**″, **6**″), 92.3 CH (**7**), 89.5 CH (**1**), 73.1 CH (**5**), 72.0 CH (**3**), 69.0 CH (**2**), 63.0 CH_2_ (**4**), 62.0 CH (**6**), 55.4 CH_3_ (**OCH_3_**), 30.9 CH_3_ (**Ph-CH_3_**), 26.6–20.1 (5 **acetyl-CH_3_**). IR: υ (cm^–1^): 1755, 1737, 1667 (C=O), 1221 (C–O). Theoretical HRMS (M + Na)^+^ = 662.23258, Exp. *m*/*z* = 662.23283.

#### 3.7.5. 2-(3-Acetyl-1-(3-chlorophenyl)-5-(4-methoxyphenyl)-1,2,4-triazolo-4-yl)-2-deoxy-1,3,4,6-tetraacetyl-β-ᴅ-glucose (**8e**)

The product comprised large yellow blocks (68.5% yield) with mp 141–142 °C and optical rotation: ${\left[\propto \right]}_{589}^{25}$ +48.29 (ethyl acetate).

^1^H NMR (700 MHz, CDCl_3_) δ (ppm): 7.25 (d, *J* = 8.73 Hz, 2H, **2**′, **6**′), 6.99 (t, *J* = 8.10 Hz, 1H, **5**″), 6.97 (t, *J* = 2.10 Hz, 1H, **2**″), 6.86 (d, *J* = 8.85 Hz, 2H, **3**′, **5**′), 6.77 (m, 1H, **6**″), 6.58 (m, 1H, **4**″), 6.50 (d, *J* = 8.36 Hz, 1H, **1**), 5.93 (s, 1H, **7**), 5.47 (m, 1H, **3**), 4.96 (dd, *J* = 9.55, 10.00 Hz, 1H, **4**), 4.27 (dd, *J* = 12.55, 4.60 Hz, 1H, **6_a_**), 3.97 (dd, *J* = 12.40, 2.10 Hz, 1H, **6_b_**), 3.85 (m, 1H, **5**), 3.73 (s, 3H, **OCH_3_**), 3.16 (dd, *J* = 9.26, 9.45 Hz, 1H, **2**), 2.51–1.93 (5 singlets, **acetyl–CH_3_**). ^13^C NMR (176 MHz, CDCl_3_) δ (ppm): 191.9–168.6 (5 **carbonyl carbons**), 160.8 C (**4′**), 135.1 C (**8**), 132.0 C (**1**″), 130.4 C (**3**″), 130.0 C (**1**′), 125.6 CH (**5**″), 128.6 CH (**2**′, **6**′), 123.5 CH (**4**″), 121.2 C (**6**″), 114.7 CH (**3**′, **5**′), 111.6 CH (**2**″), 92.1 CH (**7**), 88.8 CH (**1**), 73.0 CH (**5**), 72.0 CH (**3**), 68.7 CH (**2**), 62.6 CH_2_ (**4**), 61.6 CH (**6**), 55.4 OCH_3_ (**5′**), 29.6–20.4 (5 **acetyl–CH_3_**). IR: υ (cm^–1^): 1756, 1736, 1680 (C=O), 1211 (C–O). Theoretical HRMS (M + Na)^+^ = 682.17796, Exp. *m*/*z* = 682.17771.

#### 3.7.6. 2-(3-Acetyl-1-(4-bromophenyl)-5-((S)-4-chlorophenyl)-1,2,4-triazolo-4-yl)-2-deoxy-1,3,4,6-tetraacetyl-β-ᴅ-glucose (**8f**)

The product comprised shiny greenish-yellow needles (45.4% yield) with mp 146–147 °C and optical rotation: ${\left[\propto \right]}_{589}^{25}$ − 29.33 (ethyl acetate).

^1^H NMR (700 MHz, CDCl_3_) δ (ppm): 7.43 (d, *J* = 8.40 Hz, 2H, **3**″, **5**″), 7.37 (d, *J* = 8.40 Hz, 2H, **3**′, **5**′), 7.30 (m, 4H, **2**′, **6**′ & **2**″, **6**″), 6.72 (d, *J* = 8.65 Hz, 1H, **1**), 6.13 (s, 1H, **7**), 5.46 (dd, *J* = 8.65, 8.87 Hz, 1H, **3**), 4.96 (dd, *J* = 9.50, 9.89 Hz, 1H, **4**), 4.28 (m, 1H, **5**), 3.97 (dd, *J* = 12.44, 2.00 Hz, 1H, **6_a_**), 3.87 (m, 1H, **6_b_**), 3.16 (m, 1H, **2**), 2.58–1.62 (5 singlets, 5 **acetyl–CH_3_**). ^13^C NMR (176 MHz, CDCl_3_) δ (ppm): 190.71–168.45 (5 **carbonyl carbons**), 140.59 C (**8**), 137.73 C (**4**′), 135.96 C (**1**″), 132.10 CH (**3**″, **5**″), 129.81 C (**4**″), 129.63 CH (**2**′, **6**′), 128.81 CH (**3**′, **5**′),115.30 CH (**2**″, **6**″), 114.20 C (**1**′), 93.12 CH (**7**), 88.60 CH (**1**), 72.94 CH (**3**), 72.06 CH (**2**), 68.78 CH (**4**), 61.18 CH_2_ (**6**), 20.92–20.58 (5 **acetyl–CH_3_**). IR: υ (cm^–1^): 1745, 1688 (C=O), 1218 (C–O). Theoretical HRMS (M + Na)^+^ = 730.07790, Exp. *m*/*z* = 730.07765.

#### 3.7.7. 2-(3-Acetyl-1-(p-tolyl)-5-((R)-4-chlorophenyl)-1,2,4-triazolo-4-yl)-2-deoxy-1,3,4,6-tetraacetyl-β-ᴅ-glucose (**8g**)

The product was purified using prep. TLC to yield a greenish-yellow powder (75% yield) with mp 146–148 °C and optical rotation: ${\left[\propto \right]}_{589}^{25}$ − 34.04 (DMSO).

^1^H NMR (700 MHz, CDCl_3_) δ (ppm): 7.33 (d, *J* = 8.50 Hz, 1H, **2**′), 7.29 (m, 1H, **6**′), 7.26 (m, 1H, **3**′), 6.92 (m, 2H, **3**″, **5**″), 6.85 (m, 1H, **5**′), 6.75 (m, 1H, **2**″), 6.72 (m, 1H, **6**″), 6.52 (d, *J* = 8.45 Hz, 1H, **1**), 5.96 (d, *J* = 17.61 Hz, 1H, **7**), 5.46 (dd, *J* = 19.92, 10.00 Hz, 1H, **3**), 4.96 (dd, *J* = 9.56, 10.02 Hz, 1H, **4**), 4.28 (m, 1H, **5**), 3.97 (dd, *J* = 12.42, 2.00 Hz, 1H, **6_a_**), 3.87 (m, 1H, **6_b_**), 3.14 (m, 1H, **2**), 2.49 (d, *J* = 0.9 Hz, 3H, **Ph–CH_3_**), 2.16–1.59 (5, 5 **acetyl–CH_3_**). ^13^C NMR (176 MHz, CDCl_3_) δ (ppm): 190.71–169.51 (5 **carbonyl carbons**), 160.59 C (**8**), 138.47 C (**4**′), 135.59 C (**1**″), 130.31 C (**1**′), 129.79 CH (**3**″, **5**″, **4**″), 129.66 C (**3**′, **5**′), 129.46 CH (**2**′, **6**′), 128.61, 125.96 CH (**2**″, **6**″), 92.25 CH (**7**), 89.12 CH (**1**), 73.08 CH (**5**), 72.00 CH (**3**), 68.82 CH (**2**), 61.66 CH (**4**), 55.41 CH_2_ (**6**), 26.58 (**Ph–CH_3_**), 20.79–20.39 (5 **acetyl-CH_3_**). IR: υ (cm^–1^): 1748, 1733, 1669 (C=O), 1218 (C–O). Theoretical HRMS (M + Na)^+^ = 666.18304, Exp. *m*/*z* = 666.18244.

#### 3.7.8. 2-(3-Acetyl-1-(naphthalen-1-yl)-5-(4-methylphenyl)-1,2,4-triazolo-4-yl)-2-deoxy-1,3,4,6-tetraacetyl-β-ᴅ-glucose (**8h**)

The product comprised shiny yellow microcrystals (48% yield) with mp 148–150 °C and optical rotation: ${\left[\propto \right]}_{589}^{25}$ − 29.33 (ethyl acetate).

^1^H NMR (700 MHz, CDCl_3_) δ (ppm): 7.78 (dd, *J* = 8.24, 1.50 Hz, 1H, **4n**), 7.54 (d, *J* = 8.24 Hz, 1H, **3n**), 7.48 (m, 2H, **5n**, **6n**), 7.33 (m, 2H, **2**′, **6**′), 7.23 (m, 2H **8n**, **9n**), 6.87 (d, *J* = 7.54 Hz, 1H, **10n**), 6.79 (d, *J* = 8.30 Hz, 2H, **3′**, **5′**), 6.66 (d, *J* = 8.33 Hz, 1H, **1**), 6.42 (s, 1H, **7**), 5.67 (dd, *J* = 10.11, 10.20 Hz, 1H, **3**), 5.07 (dd, *J* = 9.54, 8.92 Hz, 1H, **4**), 4.05 (m, 1H, **5**), 4.01 (dd, *J* = 2.10, 12.01 Hz, 1H, **6_a_**), 3.87 (m, 1H, **6_b_**), 3.72 (s, 3H, **OCH_3_**), 3.25 (dd, *J* = 9.52, 9.05 Hz, 1H, **2**), 2.55–1.58 (5 **acetyl-CH_3_**). ^13^C NMR (176 MHz, CDCl_3_) δ (ppm): 189.98–167.76 (5 **carbonyl carbons**), 162.67–108.09 (**Aromatic carbons**), 147.70 C (**8**), 91.29 CH (**7**), 89.68 CH (**1**), 72.24 CH (**3**), 70.90 CH (**2**), 62.43 CH (**4**), 60.61 CH_2_ (**6**), 54.26 (**5′**), 30.90–13.18 (5 **acetyl–CH_3_**). IR: υ (cm^−1^): 1743, 1733, 1655 (C=O), 1214 (C–O). Theoretical HRMS (M + Na)^+^ = 698.23258, Exp. *m*/*z* = 698.23284.

#### 3.7.9. 2-(3-Acetyl-1-(phenyl)-5-((R)-p-tolyl)-1,2,4-triazolo-4-yl)-2-deoxy-1,3,4,6-tetraacetyl-β-ᴅ-glucose (**8i**)

The product comprised greenish-yellow cubic crystals (60% yield) with mp 148–150 °C.

^1^H NMR (700 MHz, CDCl_3_) δ (ppm): 7.23 (m, 2H, **2**′, **6**′), 7.14 (d, *J* = 7.84 Hz, 2H, **3**″, **5**″), 7.11 (m, 2H, **3**′, **5**′), 6.86 (m, 2H, **2**″, **6**″), 6.81 (m, 1H, **4**″), 6.53 (d, *J* = 8.38 Hz, 1H, **1**), 5.97 (s, 1H, **7**), 5.47 (dd, *J* = 10.09, 10.41 Hz, 1H, **3**), 4.97 (m, 1H, **4**), 4.28 (dd, *J* = 12.41, 4.52 Hz, 1H, **6_a_**), 3.97 (dd, *J* = 12.40, 2.11 Hz, 1H, **6_b_**), 3.88 (m, 1H, **5**), 3.18 (m, 1H, **2**), 2.50 (s, 3H, **Ph–CH_3_**), 2.27–1.54 (5 singlets, 5 **acetyl–CH_3_**). ^13^C NMR (176 MHz, CDCl_3_) δ (ppm): 190.7–168.7 (5 **carbonyl carbons**), 144.9 C (**8**), 142.2 C (**4**′), 139.6 C (**1**″), 136.8 C (**4**″), 129.9 CH (**3**″, **5**″), 129.1 CH (**3**′, **5**′), 121.4 CH (**1**′), 127.2 CH (**2**′, **6**′), 113.8 CH (**2**″, **6**″), 92.2 CH (**1**), 89.4 CH (**7**), 73.0 CH (**3**), 72.0 CH (**5**), 68.9 CH (**4**), 63.1 CH_2_ (**6**), 61.7 (**2**), 26.7 CH_3_ (**Ph–CH_3_**), 21.8–20.1 (5 **acetyl–CH_3_**). IR: υ (cm^–1^): 1745, 1739, 1671 (C=O), 1211 (C–O). Theoretical HRMS (M + Na)^+^ = 632.22201, Exp. *m*/*z* = 632.22206.

#### 3.7.10. 2-(3-Acetyl-1-(3,4-dichlorophenyl)-5-(3-methylphenyl)-1,2,4-triazolo-4-yl)-2-deoxy-1,3,4,6-tetraacetyl-β-ᴅ-glucose (**8j**)

The product comprised shiny yellow microcrystals with 32% yield with mp 155–156 °C and optical rotation: ${\left[\propto \right]}_{589}^{25}$ + 3.72 (ethyl acetate).

^1^H NMR (700 MHz, CDCl_3_) δ (ppm): 7.30 (m, 1H, **3′**), 6.92 (dd, *J* = 7.77, 1.14 Hz, 1H, **2′**), 6.87 (m, 1H, **4**′), 6.79 (d, *J* = 8.64 Hz, 1H, **6**′), 6.78 (t, *J* = 1.82 Hz, 1H, **3**″), 6.71 (d, *J* = 1.76 Hz, 1H, **6**′), 6.68 (s, 1H, **2**″), 6.49 (d, *J* = 8.32 Hz, 1H, **1**), 5.89 (s, 1H, **7**), 4.97 (dd, *J* = 8.66, 10.04 Hz, 1H, **3**), 4.27 (dd, *J* = 4.55, 12.42 Hz, 1H, **4**), 4.22 (m, 1H, **5**), 3.97 (dd, *J* = 12.42, 2.20 Hz, 1H, **6_a_**), 3.76 (m, 1H, **6_b_**), 3.74 (s, 3H, **OCH_3_**), 3.19 (m, 1H, **2**), 2.52–1.50 (5 singlets, 5 **acetyl-CH_3_**). ^13^C NMR (176 MHz, CDCl_3_) δ (ppm): 190.90–168.63 (5 **carbonyl carbons**), 160.58 C (**5**′), 145.77 C (**1**″), 143.68 C (**8**), 140.04 C (**1**′), 135.49 C (**3**″, **5**″), 130.77 CH (**2**′, **4**′), 120.85 CH (**3**′), 119.46 CH (**5**′), 115.38 CH (**4**″),111.98 CH (**2**″, **6**″), 93.28 CH (**7**), 92.03 CH (**1**), 72.99 CH (**3**), 72.10 CH (**2**), 68.76 CH (**4**), 61.60 CH_2_ (**6**), 55.41 CH_3_ **(OCH_3_**), 27.31–20.18 (5 **acetyl-CH_3_**). IR: υ (cm^–1^): 1740, 1680 (C=O), 1214 (C–O). Theoretical HRMS (M + Na)^+^ = 716.13898, Exp*. m*/*z* = 716.13720.

## 4. Conclusions

Ten members of a new series (**8a**–**j**) of variously substituted 4,5-dihydro-1*H*-[1,2,4] triazoline derivatives were synthesized via a 1,3-dipolar cycloaddition reaction using glucopyranose as a chiral scaffold. To the best of our knowledge, the synthetic pathway employed in this work was the first example to construct a triazoline ring on a carbohydrate moiety. The chemical identities of the derivatives were established through high-resolution mass spectrometry and vibrational spectroscopy. The solution structures were characterized through NMR spectroscopic techniques. The single-crystal X-ray analysis of derivative **8b** confirmed the 3-D structure of this molecule. Moreover, the crystal structure revealed the absolute (*S*) configuration of the newly generated stereo-center (C-7). The absolute configuration of the stereogenic center (C-7) for each of the other crystallographically characterized derivatives **8a**, **c**, **f**, **g**, and **i** was determined in a separate study, which will be reported elsewhere after DFT calculations. The X-ray structure of **8b** has shown extensive intermolecular and intramolecular forces. The nature and extent of such non-covalent interactions in the crystal lattices of the members of this series of chiral compounds could play a significant role in the absolute configuration adopted by the newly generated stereo-center (C-7). Selected derivatives were evaluated for their anti-cancer activity against mainly nine different cancer types, namely leukemia, non-small cell lung cancer, colon cancer, CNS cancer, melanoma cancer, ovarian cancer, renal cancer, prostate cancer, and breast cancer. Derivative **8c** showed the highest overall anti-tumor activity of 58.86% growth inhibition and provided better results against leukemia, CNS cancer, renal cancer, and breast cancer. The anti-microbial activity of all investigated compounds showed promising inhibition results; a remarkable anti-fungal activity for halogen and methoxy substituted compounds was observed against *A. flavus*, *A. ochraceus*, *P. citrus*, and *P. oxalicum*.

## Figures and Tables

**Table 1:** Crystallographic data for derivative **8b**.

Empirical formula	C_31_H_34_BrN_3_O_11_
Molar mass (g/mol)	704.52
Temperature (K)	298.15
Crystal system	monoclinic
Space group	*P*2_1_
*a* (Å)	11.850(2)
*b* (Å)	10.170(2)
*c* (Å)	16.090(3)
α (°)	90
β (°)	104.40(3)
γ (°)	90
Volume (Å^3^)	1878.2(7)
*Z*	2
ρ_calc (_g/cm^3^)	1.246
*μ* (mm^–1^)	1.149
*F* (000)	728.0
Crystal size (mm)	0.10 × 0.10 × 0.05
Radiation	Mo-Kα (λ = 0.71073 Å)
2*Θ* range for data collection (°)	4.904–49.414
Index ranges	−13 ≤ *h* ≤ 13, −11 ≤ *k* ≤ 11, −18 ≤ *l* ≤ 18
Reflections collected	12,658
Independent reflections	6077 [*R*_int_ = 0.0798, *R*_sigma_ = 0.0869]
Data/restraints/parameters	6077/355/418
Goodness of fit on *F*^2^	1.015
Final *R* indexes [*I* ≥ 2σ (*I*)]	*R*_1_ = 0.0722, *wR*_2_ = 0.1646
Final *R* indexes [all data]	*R*_1_ = 0.1433, *wR*_2_ = 0.2008
Largest diff. peak/hole (e Å^–3^)	0.24/−0.35

**Table 2:** Selected H-bonding interactions (Å, °) in derivative **8b**.

*D*–H···*A*	*D*–H	H···*A*	*D*–*A*	*D*–H···*A*	Symmetry Codes
C(3)–H(3)···O(10)	0.98	2.48	3.163(12)	126.7	
C(5)–H(5)···O(10)	0.98	2.30	3.046(12)	132.3	
C(8)–H(8C)···O(10)	0.96	2.66	3.48(2)	143.1	−*x* + 1, y + ½, −*z* + 1
C(10)–H(10B)···O(9)	0.96	2.59	3.404(14)	142.2	*x* + 1, *y*, *z*
C(20)–H(20)···O(3)	0.93	2.59	3.481(16)	160.3	−*x* + 1, y + ½, −*z* + 1
C(12)–H(12B)···Br(1)	0.96	2.94	3.89(2)	170.0	−*x* + 1, y−½, −*z*

**Table 3:** Selected bond distances (Å) for derivative **8b**.

C(29)–Br(1)	1.887(13)
C(16)–O(10)	1.189(13)
C(15)–N(1)	1.415(13)
C(15)–N(2)	1.299(12)
N(2)–N(3)	1.361(11)
C(18)–N(1)	1.490(12)
C(18)–N(3)	1.505(13)
C(18)–C(19)	1.485(14)
C(7)–O(2)	1.340(14)
C(7)–O(3)	1.165(17)
C(9)–O(4)	1.358(11)
C(9)–O(5)	1.174(13)
C(11)–O(6)	1.343(14)
C(11)–O(7)	1.186(15)
C(13)–O(8)	1.341(13)
C(13)–O(9)	1.217(14)

**Table 4:** The mean growth percent of treated cells (G%) values for all investigated samples.

Compound ID	NCI Code	Mean G%
**8a**	835808	92.73
**8b**	835811	70.77
**8c**	835812	58.86
**8d**	835813	78.32
**8e**	835815	73.52
**8f**	835816	61.24

**Table 5:** Screening for anti-tumor activity of **8c** and **8f** against selected cell lines. Results are presented as growth percentage (G%).

Cancer Panel Type	Cancer Cell Type	G%	
		8c NCI Code 835812	8f NCI Code 835816
Leukemia	HL-60(TB)	14.42	34.14
	RPMI-8226	35.38	38.03
	MOLT-4	41.79	44.34
CNS Cancer	SF-268	58.40	58.57
	SF-295	29.43	36.96
Renal Cancer	786-0	68.84	58.46
	A498	59.58	131.61
	ACHN	52.23	50.09
	UO-31	32.19	27.59
Breast Cancer	MCF7	43.90	34.59
	T-47D	26.78	34.57
	MDA-MB-468	49.38	26.20

## Data Availability

Data are contained within the article.

## Supplementary Materials

The following supporting information can be downloaded at: https://www.mdpi.com/article/10.3390/molecules29122839/s1, and CCDC 2327704 contains the supplementary crystallographic data for 2-(3-acetyl-1-(4-bromophenyl)-5-((S)-4-methoxyphenyl)-1,2,4-triazolo-4-yl)-2-deoxy-1,3,4, 6-tetraacetyl-β-ᴅ-glucose (**8b**) in this paper. These data can be obtained free of charge at http://www.ccdc.cam.ac.uk/data_request/cif (accessed on 2 June 2024), by emailing data_request@ccdc.cam.ac.uk, or by contacting the Cambridge Crystallographic Data Centre, 12 Union Road, Cambridge CB2 1EZ, UK; fax: +44-1223-336033. Figure S1. HSQC NMR spectrum for compound 8a in CDCl3. Figure S2. HMBC NMR spectrum for compound **8a** in CDCl3. Figure S3. A 1D chain of hydrogen-bonded **8b** molecules along the b axis. Figure S4. FTIR spectrum of derivative **8a**.
