# Supplementary material for: Stereoselective Asymmetric Syntheses of Molecules with a 4,5-Dihydro-1H-[1,2,4]-Triazoline Core Possessing an Acetylated Carbohydrate Appendage: Crystal Structure, Spectroscopy, and Pharmacology
Source: Molecules. 2024 Jun 14;29(12):2839. doi: 10.3390/molecules29122839 (PMC11206253; doi:10.3390/molecules29122839)
Supplement: Supplementary file 1 [file molecules-29-02839-s001.zip › molecules-3030759-supplementary.pdf]

## SUPPLEMENTARY MATERIALS

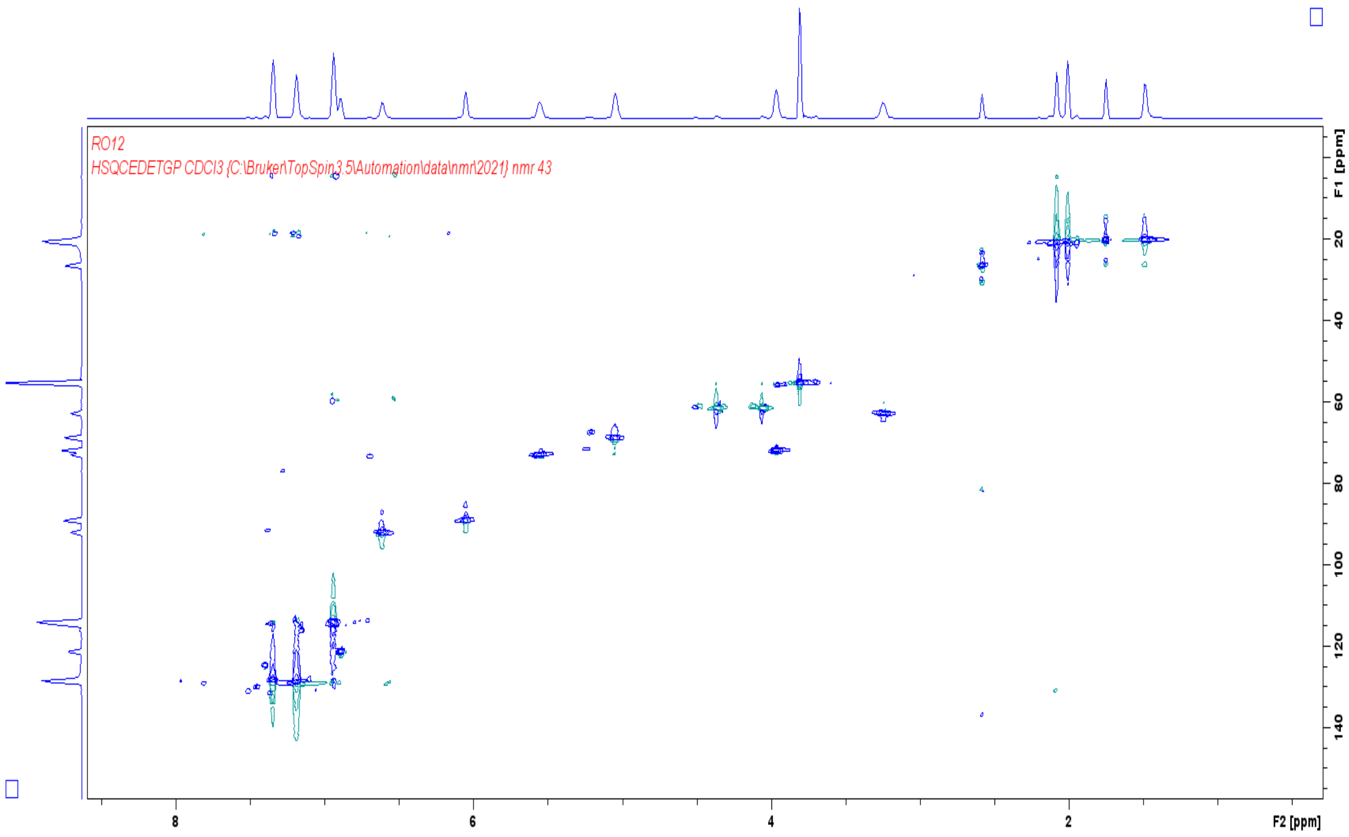


**Figure S1.** HSQC NMR spectrum for compound **8a** in $CDCl_3$.

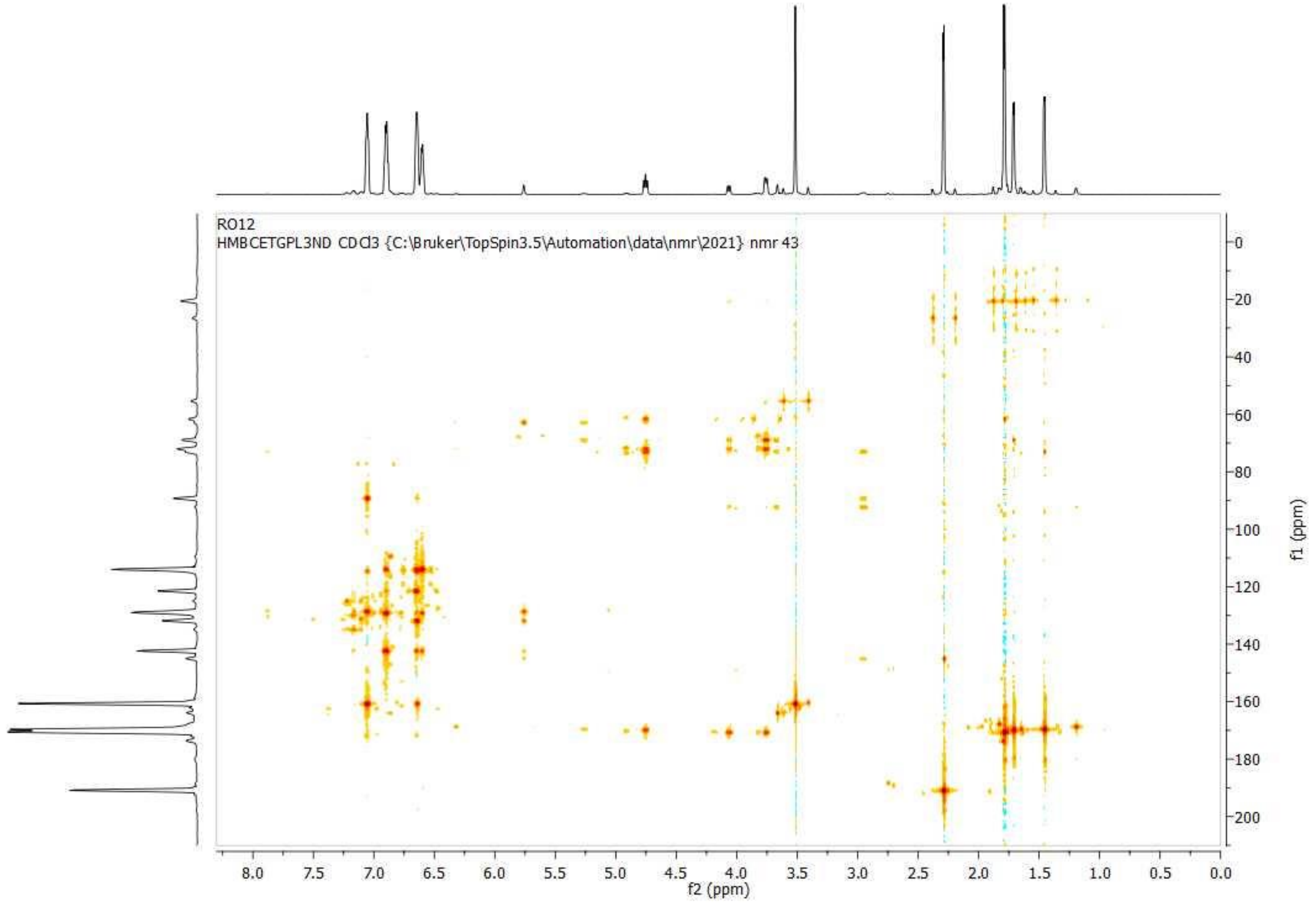


**Figure S2.** HMBC NMR spectrum for compound **8a** in $CDCl_3$.

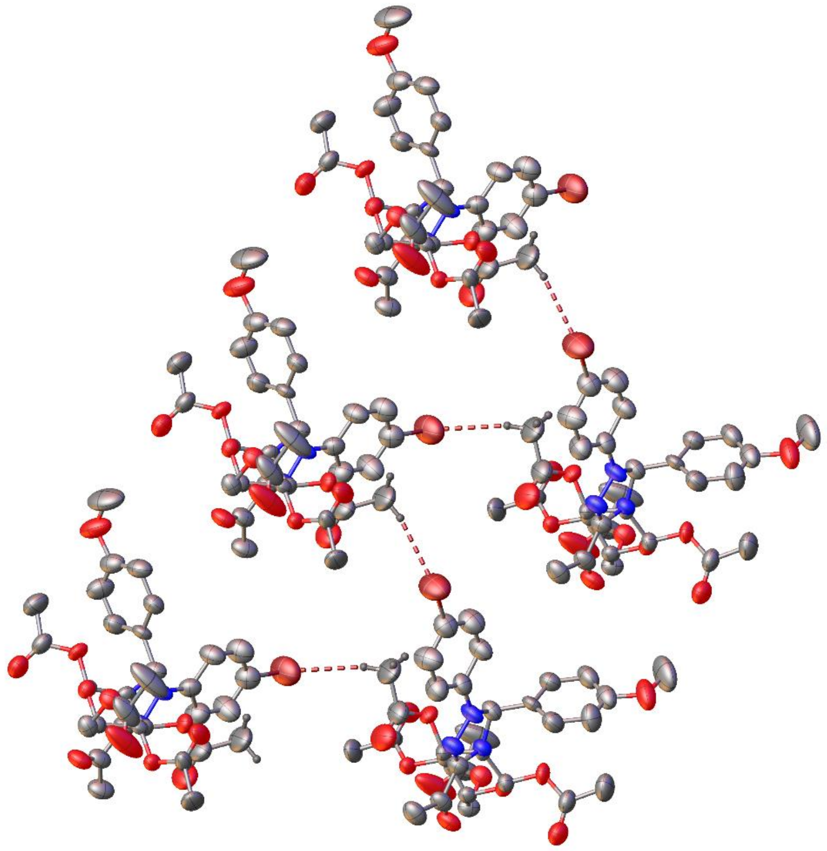

**Figure S3.** A 1D chain of hydrogen-bonded **8b** molecules along the *b* axis.

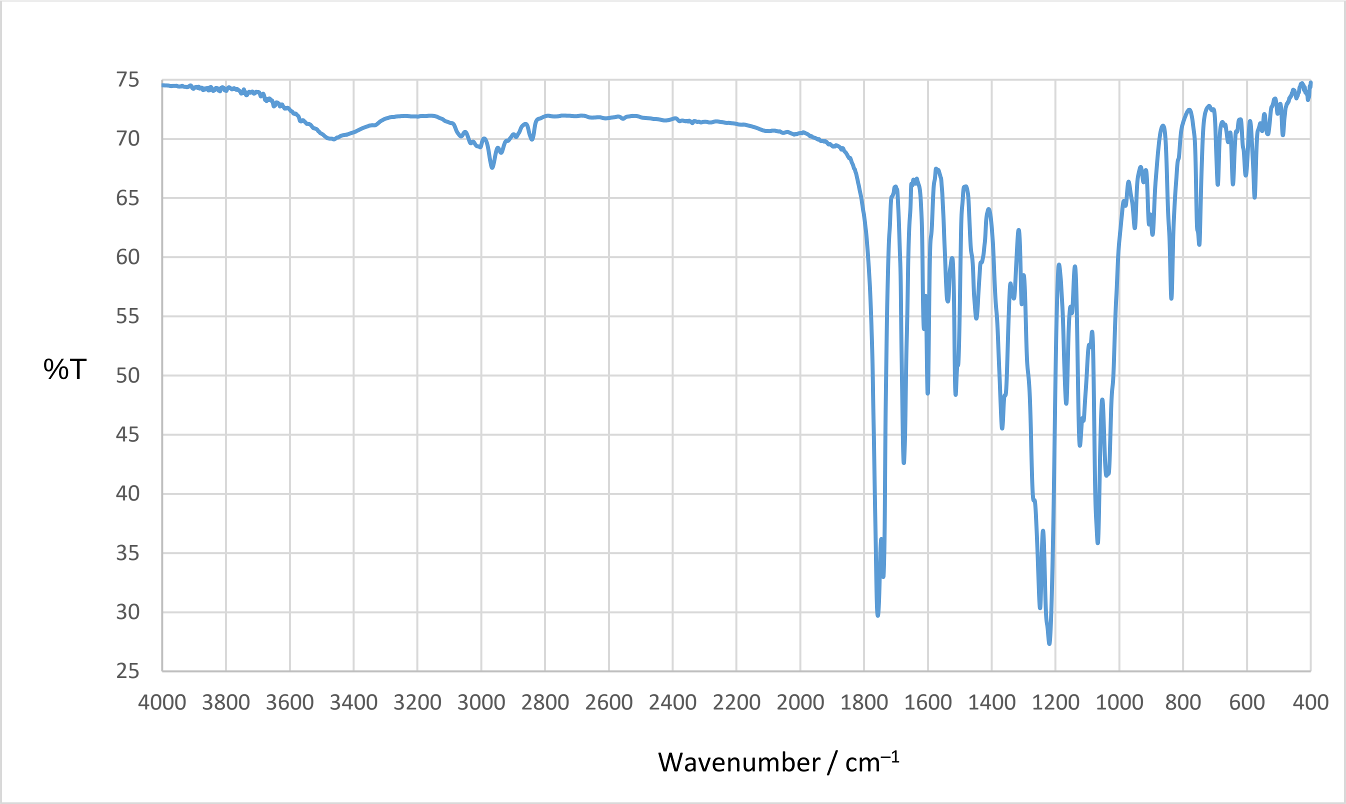


**Figure S4.** FTIR spectrum of derivative **8a**.
